# Factors associated with non-initiation of latent tuberculosis treatment among healthcare workers with a positive interferon-gamma releasing assay

**DOI:** 10.1038/s41598-018-37319-7

**Published:** 2019-01-11

**Authors:** Hyun Lee, Gun Woo Koo, Ji-Hee Min, Tai Sun Park, Dong Won Park, Ji-Yong Moon, Sang-Heon Kim, Tae Hyung Kim, Ho Joo Yoon, Jang Won Sohn

**Affiliations:** Department of Internal Medicine, Hanyang University College of Medicine, Seoul, Korea

## Abstract

Despite widespread use of the interferon-gamma release assay for the diagnosis of latent tuberculosis infection (LTBI), the initiation rate of and factors associated with LTBI treatment among healthcare workers (HCWs) have not been studied in depth. The aim of this study was to evaluate the initiation rate of LTBI treatment and also to identify any factors associated with non-initiation of LTBI treatment among HCWs. A retrospective cohort study of 293 HCWs with LTBI was performed at a teaching hospital in Korea. LTBI was diagnosed using QuantiFERON-TB Gold In-Tube tests (Cellestis Ltd., Carnegie, VIC, Australia). Of the 293 HCWs with LTBI, 189 HCWs (64.5%) visited an outpatient clinic for a medical consultation regarding LTBI treatment. Of these, 128 (67.7%) consented to LTBI treatment for a 43.7% LTBI treatment initiation rate. Upon multivariable analysis, having a liver disease or currently taking hepatotoxic drugs (adjusted odds ratio [OR] = 12.03, 95% confidence interval [CI] = 3.12–46.35), being a physician (adjusted OR = 14.01, 95% CI = 2.82–69.74) and other patient-related HCWs (adjusted OR = 3.58, 95% CI = 1.46–8.78), and years of employment ≥20 years (adjusted OR = 4.77, 95% CI = 1.74–13.12) were independent factors associated with the non-initiation of LTBI treatment. Upon bivariate multivariable analysis, while having a liver disease or currently taking hepatotoxic drugs (adjusted OR = 12.85, 95% CI = 3.06–55.92), being a physician (adjusted OR = 28.43, 95% CI = 4.78–169.28) and other patient-related HCWs (adjusted OR = 4.80, 95% CI = 1.56–14.74), and years of employment ≥20 years (adjusted OR = 4.55, 95% CI = 1.37–15.15) were factors associated with no outpatient clinic visit for a consultation of LTBI treatment, having a liver disease or currently taking hepatotoxic drugs (adjusted OR = 11.76, 95% CI = 2.68–51.73) and years of employment ≥20 years (adjusted OR = 5.29, 95% CI = 1.38–20.19) were factors associated with refusal of LTBI treatment after a consultation. The overall initiation rate of LTBI treatment was suboptimal in HCWs with LTBI diagnosed using an interferon-gamma releasing assay. Having a liver disease or currently taking hepatotoxic drugs, being a physician and other patient-related HCWs, and years of employment ≥20 years were associated with non-initiation of LTBI treatment.

## Introduction

Healthcare workers (HCWs) are at higher risk of *M. tuberculosis* infection due to their risk of occupational exposure to patients with active pulmonary tuberculosis^1,2^. Worldwide, the latent tuberculosis infection (LTBI) rate among HCWs was reported to range from 4–64%; this rate varied widely according to the burden of the study population^3,4^. Recent studies have shown that the LTBI rate among HCWs in Korea, an intermediate-tuberculosis (TB) burden country, was from 15–37%^5–10^.

The successful treatment of LTBI among HCWs is important for two clinical reasons. First, it can prevent HCWs with LTBI from developing into active pulmonary TB, which could be transmitted to patients. Second, treatment also improves the health of the HCWs themselves. Unfortunately, the LTBI treatment initiation rate has been suboptimal in most studies^11–13^, although a few have reported high rates^14,15^.

To enhance the treatment acceptance rate of LTBI, it is vital to identify some modifiable factors, such as the interference of prior bacilli Calmette-Guérin (BCG) vaccination, in the interpretation of the LTBI diagnosis, which significantly affects the initiation rate of LTBI treatment among HCWs^13,15^. According to previous studies, HCWs diagnosed with LTBI via a positive tuberculin skin test (TST) who had received a prior BCG vaccination were more likely to refuse LTBI treatment^13,15^.

Because the interferon gamma releasing assay (IGRA), a novel diagnostic method for LTBI, is not affected by prior BCG vaccination, IGRA is now preferred over the TST to test people who have had a prior BCG vaccination^16^. In addition, in an era of IGRA-based LTBI diagnoses, the initiation rate of and factors associated with the acceptance of LTBI treatment among HCWs might be different from those performed for LTBI diagnoses based upon the TST. However, few studies are available regarding these issues. Therefore, we aimed to evaluate the initiation rate of and factors associated with LTBI treatment initiation among HCWs.

## Results

### Study population

The baseline characteristics of the study population are summarized in Table 1. The median age was 46 years and 61.4% (180/293) were female. There were 258 patient-related HCWs including 19 physicians (6.5%), 53 nurses (18.1%), 186 other HCWs (63.5%), and 35 patient-unrelated HCWs (11.9%). The years of employment were as follows: <10 years (n = 57, 19.4%), 10–20 years (n = 67, 22.9%), and ≥20 years (n = 169, 57.7%). The common comorbidities were chronic liver diseases or currently taking hepatotoxic drugs (n = 30, 10.2%), hypertension (n = 22, 7.5%), diabetes mellitus (n = 20, 6.8%), and malignancy (n = 10, 3.4%). In addition, 34.5% of the HCWs had a previous history of working in a high-risk department. None of the HCWs tested was positive for human immunodeficiency virus infection.

**Table 1:** Baseline characteristics of the study population.

	Total (N = 293)
Age, years	46 (41–52)
21–30	21 (7.2)
31–40	44 (15.0)
41–50	129 (44.0)
>50	99 (33.8)
Sex, female	180 (61.4)
Occupation	
Patient-unrelated HCWs^*^	35 (11.9)
Patient-related HCWs	
Physicians	19 (6.5)
Nurses	53 (18.1)
Others^†^	186 (63.5)
Years of employment, years	
<10	57 (19.4)
10–20	67 (22.9)
>20 years	169 (57.7)
Comorbidities	
Liver diseases or currently taking hepatotoxic drugs	30 (10.2)
Hypertension	22 (7.5)
Diabetes mellitus	20 (6.8)
Malignancy	10 (3.4)
Immunocompromised	9 (3.1)
Others	19 (6.5)
A previous history of working in a high-risk department^‡^	
Yes	101 (34.5)
No	192 (65.5)

Data are presented as median (interquartile range) or number (%).

^*^Patient-unrelated HCWs included administrative staff, cooks, and cleaning staff. ^†^Others included radiology technicians, laboratory technicians, physical therapists, and medical technicians. ^‡^High-risk departments included internal medicine wards and outpatient clinics, isolation wards, emergency department, intensive care units, clinical laboratory, and infection-control units. HCW, heath care worker.

### The overall initiation rate of LTBI treatment among HCWs with a positive QFT-GIT and the acceptance rate of LTBI treatment among the HCWs who visited outpatient clinics

**Figure 1:**
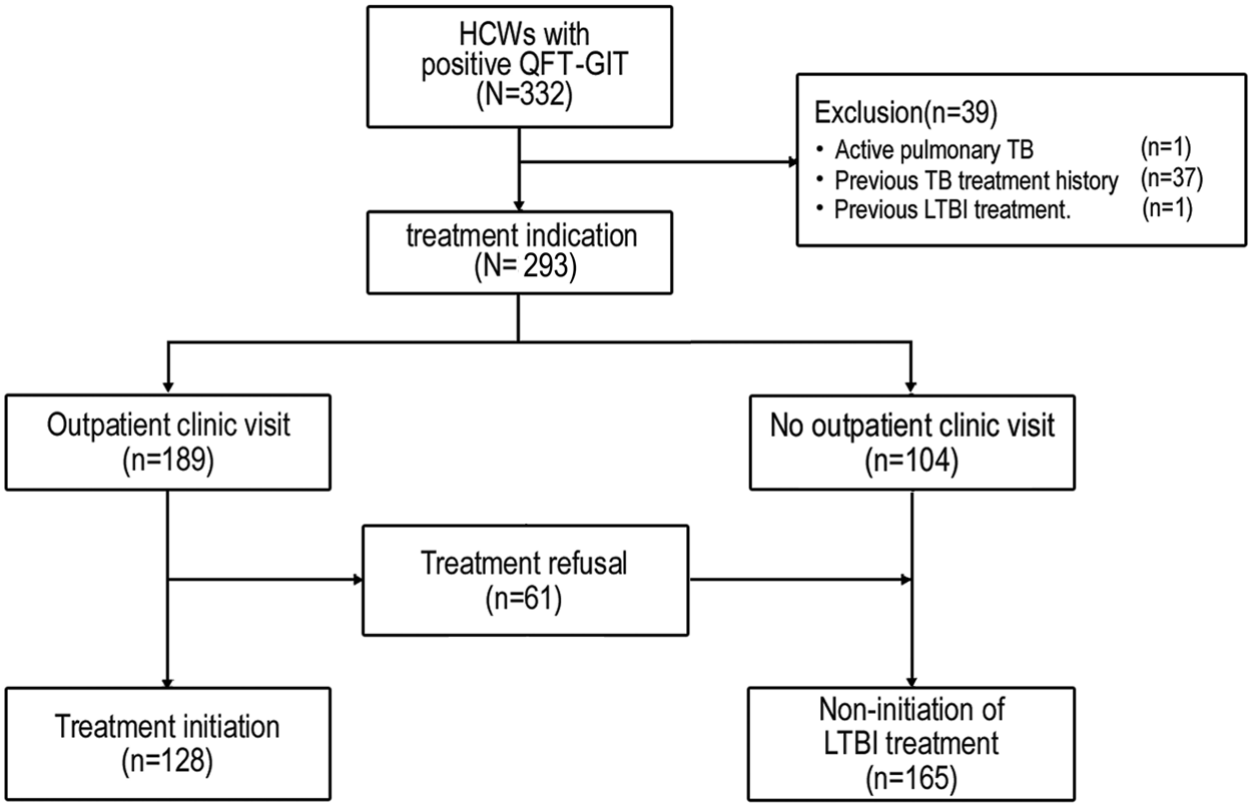
Flow chart of the study population. HCW, healthcare worker; QFT-GIT, QuantiFERON-TB In-Tube Gold test; TB, tuberculosis; LTBI, latent tuberculosis infection.

As shown in Fig. 1, the overall initiation rate of LTBI treatment was 43.7% (128/293). Of the 293 HCWs with a positive QFT-GIT, 189 (64.5%) visited an outpatient clinic for a consultation regarding LTBI treatment. Of these, 67.7% of HCWs (128/189) consented to LTBI treatment, while 32.3% (61/189) refused the LTBI treatment.

### Factors associated with non-initiation of LTBI treatment (no outpatient clinic visit or refusal of LTBI treatment) among HCWs with a positive QFT-GIT

The univariable analysis revealed that HCWs in their thirties were 2.74 (95% confidence interval [CI] = 1.01–7.40) times less likely to receive LTBI treatment compared to workers in their twenties. Physicians were 7.11 (95% CI = 1.75–28.93) times less likely to receive LTBI treatment than patient-unrelated HCWs. HCWs with a liver disease or currently taking hepatotoxic drugs were 8.15 (95% CI = 2.41–27.53) times less likely to receive LTBI treatment than those without a liver disease or currently taking hepatotoxic drugs. HCWs who had been employed for more than 20 years were 2.31 (95% CI = 1.26–4.26) times less likely to receive LTBI treatment compared to those who had worked for less than 10 years. In the multivariable analysis, HCWs with a liver disease or currently taking hepatotoxic drugs were 12.03 (95% CI = 3.12–46.35) times less likely to receive LTBI treatment than those without a liver disease or currently taking hepatotoxic drugs. Physicians and patient-related other HCWs were found to be 14.01 (95 CI = 2.82–69.74) and 3.58 (95% CI = 1.46–8.78) times less likely to receive LTBI treatment compared to patient-unrelated HCWs, respectively. In addition, HCWs with years of employment >20 years were 4.77 (95% CI = 1.74–13.12) times less likely to receive LTBI treatment compared to those with years of employment <10 years (Table 2).

**Table 2:** Unadjusted and adjusted odds ratios of the clinical variables associated with non-initiation of LTBI treatment among the HCWs with LTBI.

	Total (N = 293)	Initiation of LTBI treatment		Unadjusted OR (95% CI)	Adjusted OR (95% CI)
		Yes (n = 128)	No (n = 165)		
Age group, years					
21–30	25 (8.5)	15 (11.7)	10 (6.1)	Reference	Reference
31–40	48 (16.4)	17 (13.3)	31 (18.8)	2.74 (1.01–7.40)	1.58 (0.41–6.11)
41–50	131 (44.7)	62 (48.4)	69 (41.8)	1.67 (0.7–3.99)	0.60 (0.16–2.26)
>50	89 (30.4)	34 (26.6)	55 (33.3)	2.43 (0.98–6.01)	0.65 (0.16–2.64)
Sex					
Male	113 (38.6)	45 (35.2)	68 (41.2)	Reference	Reference
Female	180 (61.4)	83 (64.8)	97 (58.8)	0.77 (0.48–1.25)	0.72 (0.40–1.28)
Occupation					
Patient-unrelated HCWs^*^	35 (11.9)	20 (15.6)	15 (9.1)	Reference	Reference
Patient-related HCWs					
Physician	19 (6.5)	3 (2.3)	16 (9.7)	7.11 (1.75–28.93)	14.01 (2.82–69.74)
Nurse	53 (18.1)	27 (21.1)	26 (15.8)	1.28 (0.54–3.03)	3.04 (1.0–9.27)
Others^†^	186 (63.5)	78 (60.9)	108 (65.5)	1.85 (0.89–3.83)	3.58 (1.46–8.78)
Comorbidities					
Liver disease or currently taking hepatotoxic drugs	30 (10.2)	3 (2.3)	27 (16.4)	8.15 (2.41–27.53)	12.03 (3.12–46.35)
Immunocompromised	9 (3.1)	3 (2.3)	6 (3.6)	1.57 (0.39–6.41)	1.84 (0.38–9.0)
Hypertension	22 (7.5)	8 (6.3)	14 (8.5)	1.39 (0.56–3.42)	1.33 (0.45–3.94)
Malignancy	10 (3.4)	6 (4.7)	4 (2.4)	0.51 (0.14–1.83)	0.32 (0.07–1.45)
Diabetes mellitus	20 (6.8)	10 (7.8)	10 (6.1)	0.76 (0.31–1.89)	0.43 (0.14–1.29)
Others	19 (6.5)	6 (4.7)	13 (7.9)	1.74 (0.64–4.71)	1.75 (0.57–5.44)
Years of employment, years					
<10	57 (19.4)	33 (25.8)	24 (14.6)	Reference	Reference
10–20	67 (22.9)	32 (25.0)	35 (21.2)	1.50 (0.74–3.06)	1.77 (0.63–4.95)
≥20	169 (57.7)	63 (49.2)	106 (64.2)	2.31 (1.26–4.26)	4.77 (1.74–13.12)
History of working in a high-risk department^‡^					
No	192 (65.5)	87 (68.0)	105 (63.6)	Reference	Reference
Yes	101 (34.5)	41 (32.0)	60 (36.4)	1.21 (0.74–1.98)	1.18 (0.65–2.15)

^*^Patient-unrelated HCWs included administrative staff, cooks, and cleaning staff.

^†^Others included radiology technicians, laboratory technicians, physical therapists, and medical technicians.

^‡^High-risk departments included internal medicine wards and outpatient clinics, isolation wards, emergency department, intensive care units, clinical laboratory, and infection-control units.

HCW, healthcare worker; LTBI, latent tuberculosis infection; OR, odds ratio; CI, confidence interval.

### Factors associated with no outpatient clinic visit for a consultation of LTBI treatment

The bivariate univariable analysis revealed that having a liver disease or currently taking hepatotoxic drugs (unadjusted odds ratio [OR] = 8.14, 95% CI = 2.32–28.63) and being a physician (unadjusted OR = 14.29, 95% CI = 3.16–64.61) were significantly associated with no outpatient clinic visit for a consultation of LTBI treatment. The bivariate multivariable analysis revealed that compared to the HCWs without a liver disease or currently taking hepatotoxic drugs, those with a liver disease or currently taking hepatotoxic drugs were 12.85 (95% CI = 3.06–55.92) times less likely to visit an outpatient clinic for a consultation of LTBI treatment. Compared to patient-unrelated HCWs, physicians or other patient-related HCWs were 28.43 (95% CI = 4.78–169.28) and 4.80 (95% CI = 1.56–14.74) times less likely to visit an outpatient clinic for a consultation of LTBI treatment. In addition, years of employment ≥20 years (unadjusted OR = 4.55, 95% CI = 1.37–15.15) were associated with no outpatient clinic visit for a consultation of LTBI treatment (Table 3).

**Table 3:** Bivariate analyses of factors associated with non-initiation of LTBI treatment (no outpatient clinic visit and refusal of LTBI treatment) according among the HCWs with LTBI.

	Visit outpatient clinics for a consultation of LTBI treatment				Refusal of LTBI treatment			
	Visit (n = 189)	No visit (n = 104)	Unadjusted OR (95% CI)	Adjusted OR (95% CI)	Acceptance (n = 128)	Refusal (n = 61)	Unadjusted OR (95% CI)	Adjusted OR (95% CI)
Age group								
21–30 years	17 (9.0)	8 (7.7)	Reference	Reference	15 (11.7)	2 (3.3)	Reference	Reference
31–40 years	27 (14.3)	21 (20.2)	2.32 (0.79–6.75)	1.03 (0.22–4.87)	17 (13.3)	10 (16.4)	4.41 (0.83–23.42)	4.01 (0.51–31.79)
41–50 years	89 (47.1)	42 (40.4)	1.27 (0.49–3.26)	0.48 (0.10–2.23)	62 (48.4)	27 (44.2)	3.27 (0.70–15.28)	0.92 (0.12–7.03)
>50 years	56 (29.6)	33 (31.7)	1.82 (0.68–4.86)	0.50 (0.10–2.57)	34 (26.6)	22 (36.1)	4.85 (1.01–23.32)	1.07 (0.55–8.63)
Sex								
Male	64 (33.9)	49 (47.1)	Reference	Reference	45 (35.2)	19 (31.1)	Reference	Reference
Female	125 (66.1)	55 (52.9)	0.61 (0.36–1.03)	0.53 0.28–1.02)	83 (64.8)	42 (68.9)	1.20 (0.62–2.30)	1.16 (0.55–2.48)
Comorbidities								
Liver disease or currently taking hepatotoxic drugs	13 (6.88)	17 (16.4)	8.14 (2.32–28.63)	12.85 (3.06–55.92)	3 (2.3)	10 (16.4)	8.17 (2.16–30.91)	11.76 (2.68–51.73)
Immunocompromised	6 (3.2)	3 (2.9)	1.24 (0.24–6.26)	1.30 (0.19–9.02)	3 (2.3)	3 (4.9)	2.15 (0.42–11.0)	2.58 (0.44–15.30)
Hypertension	11 (5.8)	11 (10.6)	1.77 (0.69–4.59)	1.71 (0.53–5.53)	8 (6.3)	3 (4.9)	0.78 (0.20–3.03)	0.79 (0.18–3.57)
Malignancy	8 (4.2)	2 (1.9)	0.40 (0.08–2.02)	0.33 (0.05–2.02)	6 (4.7)	2 (3.3)	0.69 (0.14–3.52)	0.32 (0.05–2.13)
Diabetes mellitus	14 (7.4)	6 (5.8)	0.72 (0.25–2.06)	0.36 (0.10–1.32)	10 (7.8)	4 (6.6)	0.83 (0.25–2.75)	0.50 (0.12–2.05)
Others	7 (3.7)	12 (11.5)	2.65 (0.96–7.33)	3.14 (0.97–10.16)	6 (4.7)	1 (1.6)	0.34 (0.04–2.88)	0.24 (0.02–2.31)
Occupation								
Patient-unrelated HCWs^*^	28 (14.8)	7 (6.7)	Reference	Reference	20 (15.6)	8 (13.1)	Reference	Reference
Patient-related HCWs								
Physician	4 (2.1)	15 (14.4)	14.29 (3.16–64.61)	28.43 (4.78–169.28)	3 (2.4)	1 (1.6)	0.83 (0.08–9.25)	1.56 (0.12–7.03)
Nurse	39 (20.6)	14 (13.5)	1.48 (0.51–4.34)	3.48 (0.88–13.80)	27 (21.1)	12 (19.7)	1.11 (0.38–3.22)	2.78 (0.74–10.44)
Others^†^	118 (62.5)	68 (65.4)	2.49 (0.99–6.25)	4.80 (1.56–14.74)	78 (60.9)	40 (65.6)	1.28 (0.52–3.17)	2.70 (0.94–7.75)
Years of employment, years								
<10	39 (20.6)	18 (17.3)	Reference	Reference	33 (25.8)	6 (9.8)	Reference	Reference
≥10 but <20	41 (21.7)	26 (25.0)	1.49 (0.69–3.23)	2.38 (0.71–7.92)	32 (25.0)	9 (14.8)	1.55 (0.49–4.85)	0.95 (0.22–4.01)
≥20	109 (57.7)	60 (57.7)	1.75 (0.89–3.43)	4.55 (1.37–15.15)	63 (49.2)	46 (75.4)	4.02 (1.55–10.38)	5.29 (1.38–20.19)
History of working in a high-risk department‡								
No	129 (68.3)	63 (60.6)	Reference	Reference	87 (68.0)	42 (68.9)	Reference	Reference
Yes	60 (31.7)	41 (39.4)	1.38 (0.80–2.37)	1.54 (0.79–3.0)	41 (32.0)	19 (31.1)	0.96 (0.50–1.85)	0.77 (0.36–1.68)

^*^Patient-unrelated HCWs included administrative staff, cooks and cleaning staff.

^†^Others included radiology technicians, laboratory technicians, physical therapists, and medical technicians.

^‡^High-risk departments included internal medicine wards and outpatient clinics, isolation wards, the emergency department, intensive care units, the clinical laboratory, and infection-control units.

HCW, healthcare worker; LTBI, latent tuberculosis infection; OR, odds ratio; CI, confidence interval.

### Factors associated with refusal of LTBI treatment among the HCWs who visited an outpatient clinic for a consultation of LTBI treatment

In the univariable bivariate analysis, age group >50 years (unadjusted OR = 4.85, 95% CI = 1.01–23.32), having a liver disease or currently taking hepatotoxic drugs (unadjusted OR = 8.17, 95% CI = 2.16–30.91), years of employment ≥20 years (unadjusted OR = 4.02, 95% CI = 1.55–10.38) were associated with refusal of LTBI treatment among HCWs who visited an outpatient clinic for a consultation of LTBI treatment. In the multivariable bivariate analysis, having a liver disease or currently taking hepatotoxic drugs was significantly associated with refusal of LTBI treatment among HCWs who visited an outpatient clinic for a consultation of LTBI treatment (adjusted OR = 11.76, 95% CI = 2.68–51.73). Being a HCW who worked for at least 20 years was associated with refusal of LTBI treatment (adjusted OR = 5.29, 95% CI = 1.38–20.19) (Table 3).

## Discussion

The present study evaluated factors associated with non-initiation of LTBI treatment among HCWs in a teaching hospital. Of the 293 HCWs with LTBI, about two-thirds sought medical consultation for LTBI treatment, and another two-thirds of these consented to the LTBI treatment. This study revealed that having a liver disease or currently taking hepatotoxic drugs, being a physician and other patient-related HCWs, and working in healthcare for more than 20 years were independent factors associated with non-initiation of LTBI treatment.

Before the introduction of IGRA, TST was the only modality to diagnose LTBI. However, since IGRA is known to be more specific, better correlated with the burden of TB exposure and less influenced by a prior BCG vaccination, non-tuberculous infection, and immunosuppressive treatment or immunocompromised status compared to TST^17–19^, IGRA is now widely used in many countries worldwide^20^. In the era of TST-based LTBI diagnosis, the initiation rates of LTBI treatment among HCWs varied considerably according to the study population with LTBI, which ranged between 47–98%^12,15,21,22^. While some previous studies have evaluated the use of IGRA in the diagnosis of LTBI among HCWs, these studies have focused on a comparison of the performance between IGRA and TST^5,6^. Thus, few data currently exist regarding the rate of LTBI treatment among HCWs who are diagnosed of LTBI with IGRA.

Previous studies have revealed that prior BCG vaccination was significantly associated with non-initiation of LTBI treatment among HCWs with a positive TST; a substantial proportion of HCWs with positive TSTs was regarded by physicians as having false-positive results^13^. Moreover, the HCWs who had received prior vaccinations were also more likely to refuse LTBI treatment^13,15^. The HCWs who refused LTBI treatment may have regarded their positive TST results as false-positive due to their prior BCG vaccination, or they might have had the misconception that a prior BCG vaccination would prevent LTBI from progressing to an active disease state^13^. In this view, IGRA has an advantage over TST in that it reduces the range of LTBI diagnoses arising from varied TST interpretations.

However, surprisingly, despite the aforementioned potential advantage of IGRA over TST, the initiation rate of LTBI treatment in this study was about 45% which is relatively lower compared to that in previous studies, where the LTBI was diagnosed using TST. Another study which evaluated factors associated with positive IGRA among HCWs in Korea revealed similar results^8^. However, since the previous study and our study did not compare the initiation rate of LTBI treatment between HCWs diagnosed of LTBI by IGRA versus those diagnosed by TST, future studies are needed to confirm whether diagnostic modality can affect the initiation rate of LTBI treatment.

As shown in this study, non-initiation of LTBI treatment can be caused by “no outpatient clinical visit” and “treatment refusal”. Surprisingly, in this study, the major cause of non-initiation of treatment was “no outpatient visit” rather than “treatment refusal”; about one-third of the HCWs with LTBI did not visit an outpatient clinic for a medical consultation regarding LTBI treatment, which is significantly lower compared to a previous study in which 98% of HCWs with a positive TST consulted with physicians^15^. However, since we did not interview the HCWs who did not visit an outpatient clinic, we could not identify their specific reasons for not seeking an evaluation of their condition. Despite this limitation, we found that physicians are at the highest risk of not receiving a medical consultation. In this study, only 21% of physicians visited an outpatient clinic. However, the acceptance rate of LTBI treatment among the physicians who did visit an outpatient clinic was relatively high (75%) and was in agreement with the findings of a previous study^23^. The physicians’ busier schedules might have contributed to the lower outpatient clinic visit rate. In contrast, physicians are likely more knowledgeable about LTBI than other HCWs, which might have led to a higher acceptance rate of treatment.

In this study, a longer duration of employment in the healthcare field was significantly associated with both no outpatient clinic visit and refusal of LTBI treatment. However, the fact that clinicians and healthcare workers exposed for longer duration (i.e., working for longer) refused treatment for LTBI is not surprising, considering that the predictive value of IGRA to the development of active TB is not clear and the known hepatotoxic effects of LTBI treatment win those older than 35 years is also a hindrance^24^. Another important factor associated with both no outpatient clinic visit and refusal of LTBI treatment in this study was having a liver disease or currently taking hepatotoxic drugs; this finding could be explained by the results of a previous study, which found that “believing that taking medicine would be problematic” is associated with non-acceptance of LTBI treatment initiation^11^. Thus, HCWs with a chronic liver disease or who were currently being prescribed hepatotoxic drugs might have refused LTBI treatment due to a fear of the liver toxicity often associated with LTBI treatment. The current standard or recommended anti-TB drug for LTBI for patients with a liver disease or those receiving hepatotoxic drugs has not been well established. Because first line anti-TB drugs are hepatotoxic and increase the risk of liver failure in these patients, a careful clinical judgement is necessary to ensure that the benefit of LTBI treatment outweighs the risks^24^. In addition, upon a clinical decision to treat LTBI, testing baseline liver function and regular follow-up is strongly encouraged to monitor hepatotoxicity^24^.

This study had several limitations. It was performed at a single university hospital in Korea. Since various factors, including different types of stigmas, can affect the acceptance of LTBI treatment in different ethnicities and populations, our findings might not be generalizable to HCWs in other countries. Second, we only included workers’ years of employment at our institution. As a result, HCWs who had been employed at other institutions before working at our institution would have been classified as having erroneously low years of employment. Third, we did not assess previous history of BCG vaccination of HCWs, which have been useful to assess it as a potential factor for non-initiation of LTBI treatment. Fourth, some of the 95% CIs for the results on factors associated non-initiation of LTBI treatment were quite wide probably due to relatively small number of these factors. Fifth, since we did not perform in-depth interviews with the HCWs who did not visit an outpatient clinic or who refused LTBI treatment, we could not identify specific reasons for non-initiation of LTBI treatment in at-risk HCWs. Thus, further studies that include interviews will be needed to determine some of these modifiable factors.

In conclusion, our study found that the initiation rate of LTBI treatment was suboptimal in HCWs with LTBI diagnosed using QFT-GIT; about one-third of HCWs with LTBI did not visit an outpatient clinic for a consultation regarding this condition. In addition, one-third of those who visited an outpatient clinic refused LTBI treatment. Having a liver disease or currently taking hepatotoxic drugs, being a physician and other patient-related HCWs, and having more than 20 years of employment were factors that were independently associated with non-initiation of LTBI treatment.

## Methods

### Study design and population

A retrospective cohort study to assess factors associated with non-initiation of LTBI treatment in HCWs positive for LTBI was conducted at a single university hospital between November 2016 and March 2017. During the study period, QuantiFERON-TB Gold In-Tube tests (QFT-GIT; Cellestis Ltd., Carnegie, VIC, Australia) were performed for 1,213 HCWs to screen for LTBI and were positive in 332 (27.4%) participants.

We enrolled 293 HCWs but excluded 39 who had undergone previous TB treatment (*n* = 37), had ever received LTBI treatment (*n* = 1) or were diagnosed with active pulmonary TB (*n* = 1) (Fig. 1).

The study protocol was approved by the Institutional Review Board of Hanyang University Hospital (IRB number 2017-02-018). All data were anonymized before analysis and informed consent from the study participants was waived due to the retrospective nature of this study.

### In-hospital protocol for the management of HCWs with positive QFT-GIT

All HCWs who were positive for QFT-GIT were recommended to visit an outpatient clinic of four respiratory physicians (D.W.P., S.-H.K., H.J.Y., and J.W.S.) in our institution via a standardized short message service, informing the HCW who received the message is needed to visit an outpatient clinic for a consultation regarding LTBI treatment. If a HCW with a positive QFT-GIT did not visit an outpatient clinic, the same message was sent to the HCW three times over three months at intervals of one month. When HCWs with a positive QFT-GIT visited an outpatient clinic, detailed information regarding the presence of respiratory symptoms (cough, sputum, hemoptysis, and dyspnea) and previous treatment for pulmonary TB and LTBI treatment were performed. Those with lesions suspicious of active pulmonary TB visible on chest X-rays underwent further studies to evaluate the presence of active pulmonary TB (at least two sputum acid fast stain and culture with one sputum polymerase chain reaction for the detection of *M. tuberculosis*). HCWs who were positive for one of these tests were regarded as having active pulmonary TB. Despite negative results for the above-mentioned tests, active pulmonary TB was diagnosed in HCWs when chest X-ray findings suggestive of active pulmonary TB were noticed and clinical signs were compatible with active pulmonary TB. When active pulmonary TB had been excluded, all HCWs with a positive QFT-GIT who visited an outpatient clinic were encouraged to receive LTBI treatment. The information whether a QFT-GIT positive HCW consent to or refuse LTBI treatment after consultation were all recorded in the medical chart according to in-hospital infection prevention and control guideline.

### Performance and interpretation of QFT-GIT

Two-staged QFT-GIT were performed according to the manufacturer’s instructions. One-milliliter aliquots of blood were drawn directly into three evacuated blood collection tubes: one containing heparin only (negative control), one with T cell mitogen (positive control) and one containing *M. tuberculosis*-specific antigens, including early secreted antigenic target 6, culture filtrate protein 10 and TB7.7 (TB-antigen tube). After overnight incubation, 200 ul of plasma were removed from each tube, and the concentration of interferon-gamma (IFN-γ) was measured using an enzyme-linked immunosorbent assay. A positive response was defined as an antigen-nil IFN-γ concentration ≥0.35 IU/ml^5,25^.

### Measures

At the time of LTBI screening, according to in-hospital infection prevention and control guideline, information on age, gender, type of occupation, comorbidities, hospital department, years of employment at our institution, and cellular phone number were collected using a standardized questionnaire. For the study purpose, after approval of this study by ethics committee in our institution, we merged these data with the following data collected by chart review: the results of QFT-GIT, outpatient clinic visit, and acceptance or refusal of LTBI treatment.

The HCWs’ occupations were classified as either patient-related, including physicians, nurses (nurses and nurses’ aides) and others (radiology technicians, laboratory technicians, physical therapists, and medical technicians) or patient-unrelated HCWs (administrative staff, cooks and cleaning staff) according to history of ever working. The hospital departments were divided into high-risk and low-risk departments according to their workers’ relationship with TB patient care. Accordingly, internal medicine wards and outpatient clinics, isolation wards, emergency department, intensive care units, clinical laboratory, and infection-control units were classified as high-risk departments; other departments without routine TB patients or specimen contact were classified as low-risk departments. Comorbidities included diabetes mellitus, hypertension, liver disease or currently taking hepatotoxic drugs (anti-fungal drugs, non-steroid anti-inflammatory drugs, lipid lowering agent, etc.), malignancy, immunocompromised, and others. Based on the measures of the previous studies^8–11^, age was categorized into four groups by decade: 20 s (21–30 years), 30 s (31–40 years), 40 s (41–50 years), and older (>50 years), and years of employment were categorized into three groups of <10 years, ≥10 years but <20 years (termed “10–20 years”), and ≥20 years^8–10,26^. LTBI was diagnosed in a patient who had a positive QFT-GIT.

### Statistical analysis

Data are presented as the number and percentage for categorical variables and as the median and interquartile range (IQR) for continuous variables. Categorical variables were compared using Pearson’s chi-square test or Fisher’s exact test. Continuous variables were compared using the Mann-Whitney *U* test because the assumption of normality was significant for these variables. To evaluate the factors associated with non-initiation of LTBI treatment (no outpatient clinic visit or refusal of LTBI treatment), a multivariable logistic regression analysis was performed. Considering non-initiation rate of LTBI treatment and our sample size (about 55%, n = 293), we hypothesized that nine factors can be enough for our study questions based on the study suggesting how many predictors can be derived from data when doing regression analysis^27^. Thus, a total of 11 factors entered into the logistic regression model, which included age, sex, chronic liver disease or currently taking hepatotoxic drug, immunocompromised, hypertension, malignancy, diabetes mellitus, other comorbidities, type of occupation, years of employment, and history of working in a high-risk department. We further performed bivariate analysis to evaluate the factors associated with no outpatient clinic visit and refusal of LTBI treatment. All tests were two-sided, and *P*-values < 0.05 were considered statistically significant. All statistical analyses were performed using R version 3.2.3 (R Foundation for Statistical Computing, Vienna, Austria), the Statistical Package for the Social Sciences (SPSS) for Windows (version 24.0; IBM Corp., Armonk, NY, US), and STATA (version 15.0; Stata Corporation, College Station, TX, USA).

## Data Availability

All data extracted in this study are included in this article.
